# Bilateral macular thickening in mild unilateral anterior uveitis: is HLA-B27 involved?

**DOI:** 10.1186/1471-2415-12-30

**Published:** 2012-07-29

**Authors:** Alexandra Wexler, Trond Sand, Tor B Elsås

**Affiliations:** 1 Department of Ophthalmology, St. Olavs University Hospital, Trondheim, Norway; 2 Department of Neuroscience, Norwegian University of Science and Technology, Trondheim, Trondheim, Norway; 3 Department of Neurology and Clinical Neurophysiology, St. Olavs University Hospital, Trondheim, Norway

## Abstract

**Background:**

Macular thickening (MT) without clinically recognized macular edema has been described in anterior uveitis (AU). Although fellow-eyes of patients have been used as controls in several studies, little is known about macular thickness in these eyes. We studied the rate and extent of MT in both AU-affected and quiescent fellow-eyes of phakic AU patients with good visual acuity (VA). We also assessed macular thickness related to HLA-B27 presence and to recurrence, since these issues have been almost unexplored by previous optical coherence tomography (OCT) studies.

**Methods:**

Patients with AU were prospectively included and macular thickness was measured with OCT initially and on follow up. Macular thickness in patients’ affected eyes (n = 30) as well as in their quiet fellow-eyes (n = 28) was compared with eyes of age- and gender matched controls. Inter-ocular differences in macular thickness between AU affected eyes and their fellow-eyes were assessed in patients (n = 28), also in a subgroup with visual acuity ≥ 0.8 (n = 23) by one-sample Student’s t-tests. Inter-ocular differences were also assessed related to HLA-B27 presence and related to the status of current AU episode (initial or relapse).

**Results:**

Subclinical MT is present in both quiet fellow-eyes and AU-affected eyes of patients. MT was found in most cases of AU, even in phakic eyes with good VA. There was a larger increase in macular thickness in HLA-B27-positive than in HLA-B27-negative patients. No differences in macular thickness were found between patients with their first AU episode and patients with recurrent episodes.

**Conclusions:**

MT probably reflects systemic immune-mediated response to the inflammatory disorder in AU, and it is possible that HLA-B27-related factors are involved in the pathogenesis of AU. These observations are in line with and extend the current understanding of the mechanisms behind MT in AU.

## Background

Anterior uveitis (AU) refers to a group of infectious and/or immune- mediated inflammatory disorders primary involving the iris and/or the anterior ciliary body of the eye [1,2]. A common key sign of an active AU, along with eye discomfort, is temporary reduced visual acuity (VA), whereas it is the complications of an AU that may lead to a long-lasting visual impairment.

Impairment of central vision due to persistent macular edema is the most frequent complication of uveitis, the occurrence rate of which differs for specific uveitis entities [3-5]. Although the rate of macular edema is lowest in AU, it contributes greatly to the overall visual impairment in the general population because of its high prevalence [6,7].

Macular edema involves macular thickening (MT) with or without cystoid space formation [8-10], which may be detected and objectively quantified by the optical coherence tomography (OCT) even in cases where MT escapes clinical detection [11,12]. MT may occur in the course of an AU even in eyes without posterior segment disease, and it may or may not correspond to VA or the grade of anterior chamber inflammation [11,13-15]. The duration for MT after an AU episode has not been established [16]. Although inter-eye asymmetry has been used to demonstrate MT in uveitis-affected eyes [11,13], we did not find any literature reporting macular thickness values in fellow-eyes during an uveitis episode.

HLA-B27- related AU is a distinct clinical entity with a high association with systemic rheumatic diseases [17,18]. The prevalence of the HLA-B27 antigen, its polymorphism and association strength with AU varies in different populations, the latter probably due to a combination of environmental and genetic factors [19-22]. Higher rates of macular edema and more complicated course have been reported in HLA-B27-related AU [23,24], although results differ between studies. It is feasible that not only macular edema but also MT is associated with the HLA-B27 antigen. However, although a possible association between moderate to severe acute anterior uveitis and HLA-B27 was mentioned in a previous letter [11], we found only one study comparing OCT in HLA-B27 positive vs. HLA-B27 negative AU patients [13].

For these reasons we investigated a homogeneous sample of phakic AU eyes with no clinically recognized macular edema, and their fellow-eyes by OCT in order to determine the frequency and extent of bilateral MT in hospital-based patient population. Macular thickness was also analysed with regard to the presence of HLA-B27 and by recurrence status in order to improve our understanding of the AU pathogenesis in our HLA-B27-rich population [25,26]. Our data suggest that MT also is present in quiet fellow-eyes of AU patients and that MT is rather the rule than the exception, even in phakic affected eyes with excellent visual acuity.

## Methods

Adult (>18 years old) patients with AU classified according to the primary anatomic site of the intraocular inflammation [27] were prospectively recruited from Department of Ophthalmology, St. Olavs University Hospital. Initially 38 patients consented to participate in the study. Their records were reviewed on inclusion and re-reviewed at least 6 months later in order to ascertain correct classification of the AU [2]. The study was approved by the Regional Ethics Committee in May 2005. It was conducted in accordance with the Declaration of Helsinki recommendations.

AU was defined as the presence of inflammatory cells in the anterior chamber and corneal endothelium and absence of posterior vitreous cells and/or other signs of intraocular inflammation. Inclusion criteria were AU of non-traumatic, non-infectious origin [28] without clinically recognized posterior segment involvement such as vasculitis, macular edema or optic nerve involvement seen on slit lamp biomicroscopy, best corrected VA (Humphery automatic refractor HARK 597, Dublin, CA) ≥0.2 with spheric equivalent of ±6, and intraocular pressure between 8 and 21 mmHg. Subjects with current or previous ophthalmic history other than AU, significant lens opacities, intraocular lens implants after cataract or refractive surgery, glaucoma or diagnosed diabetes were excluded.

Medical history regarding present and previous eye symptoms, their laterality, duration and treatment was recorded. For the purpose of this study time elapsed from the diagnosis of an AU flare-up was used as an approximation of time elapsed from initial AU flare-up. Symptoms of coexistent spondyloarthropathy, morning muscle stiffness and/or joint pain were asked for. The onset, duration and course of the AU and anterior chamber cells grading was managed according to Standardization of Uveitis Nomenclature [2]. Laboratory investigations were tailored individually to rule out infectious/ systemic origin/ association of AU. HLA-B27 presence was assessed in all patients, chest x-ray was performed in cases where oral corticosteroids were instituted, sacroiliac spine x-ray was performed if spondyloarthropathy was suspected, and patients with considerable systemic complaints were seen by a rheumatologist.

One patient was excluded due to amblyopic AU eye, one due to previous uveitis-related cystoid macular edema, one due to coexisting pigment dispersion syndrome, one due to abnormal pigmentation in macula of the affected eye. Three eyes of two patients were excluded due to lens implants, and another patient was excluded due to presence of posterior vitreous cells during the follow up period. Both eyes of two patients were excluded due to lens opacities and low OCT signal strength in the affected eye, leaving in total 59 included eyes of 30 patients.

All patients were treated with dexamethasone 0.1% eye drops and mydriatics according to the severity of their AU [29]. Eight subjects received a short course of oral prednisolone in addition to eye drops, because the AU did not respond promptly or adequately to initial topical treatment. Treatment was tapered down slowly after all clinical signs of AU had gone into remission. Three patients were using immunomodulatory agents which had been instituted before the actual AU episode.

All patients but one (who was Asian) were white. One patient had undergone LASIK surgery in both eyes more then 2 years prior to initial AU episode.

Data from healthy subjects reported by us earlier [30] were used as controls. Their HLA-B27 status is not known. Controls were age and gender matched to patients: mean age in female patients (n = 15, 50% females in sample) was 40.7 (SD 12.8) range 19–56. Female controls ≤53 years were included, their mean age was 40.5 (SD 9.7) range 21–53 (n = 47, 51% females in sample). Mean age in male patients (n = 15) was 40.3 (SD 13.0) range 21–69 and in male controls (n = 45, 49% males in sample) was 39.0 (SD 12.0) range 22–63. Controls were further gender-and age matched according to patient subgroups.

OCT (Optic Coherence Tomography STRATUS, Carl Zeiss Meditec, Inc., Dublin, CA) scans in macular thickness protocol (software v.5.0.1) were obtained by a single operator on inclusion and on follow up. Follow up intervals were individually tailored depending on the severity of AU, type of treatment and treatment response, as well as practicability for ophthalmologist and patient. Scans with signal strength ≥3 qualified for inclusion. Scans were qualitatively evaluated as to the presence of epiretinal membrane, vitreomacular traction, cystoid space formation and serous macular detachment. Only one scan was excluded due to cystoid space formation. Mean macular thickness was used in subsequent analyses.

**Figure 1:**
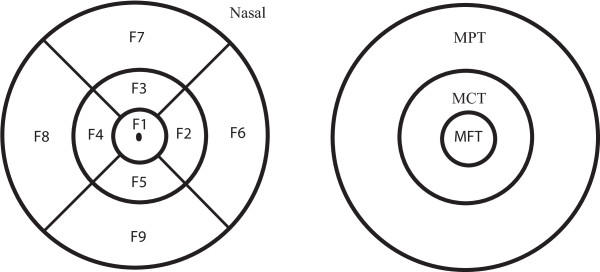
Macular areas on the Stratus OCT scan.

The macula was divided into 9 areas as described in our previous paper [30]: Mean foveal thickness (MFT = F1) from a central macular area of one millimeter in diameter, the inner ring (F2-F5) and the outer ring (F6-F9), each divided into four quadrants. The outer ring diameters measured 2.22 and 3.45 millimeters respectively. Regional variables MCT (mean central thickness) and MPT (mean peripheral thickness) were defined by averaging middle (MCT = (F2 + F3 + F4 + F5)/4) and outer (MPT = (F6 + F7 + F8 + F9)/4) ring data (Figure 1). Averaged mean total macular thickness (MTT) was calculated by averaging the nine macular areas (F1 + F2 + F3 + F4 + F5 + F6 + F7 + F8 + F9)/9. The inner region (MFT = F1) and mean minimal foveolar thickness (MMFT = F0) were also analyzed. 

In total 126 scans from both eyes in 29 patients, several of which were repeatedly taken on follow-up were available. Inter-ocular differences between AU-affected and their fellow- eyes were analyzed in relation to time elapsed from AU flare-up.

Cases were grouped in two by time elapsed from flare-up. Early measurement was defined by ≤2 weeks, late measurement by 7–12 weeks follow-up. Scans taken at 3–6 weeks and at long time remission were not included (favoring polarization). In cases where several observations of same subject were available in each group (early or late) the observation with shortest follow-up was chosen. Finally bilateral scans from 21 patients measured early and 8 patients measured late were available. Post-hoc two-sample test was performed on inter-ocular differences in macular thickness by the Mann–Whitney U-test.

For further inclusion in statistical analysis only one bilateral scan from each patient was chosen from 126 scans. In cases where several scans of a same patient were available, the scan taken 1–2 weeks after flare-up was preferred. Finally 59 scans (29 bilateral and one unilateral) from 15 males and 15 females were analyzed.

Normal MTT ranges were defined by MTT ±2SD, stratified by gender in controls; (238.35 μm - 259.33 μm) in females and (243.95 μm - 271.81 μm) in males.

Macular thickness in AU eyes of 30 patients were compared with macular thickness in gender- and age matched controls (n = 92) (one eye of each subject in both groups) by two-sample Student’s t-test. Inter-ocular differences in macular thickness between affected and fellow-eyes (n = 28) were assessed in all patients and in a subgroup with best corrected visual acuity ≥0.8 (n = 23), by one-sample Student’s t-tests (expected value = 0).

Macular thickness in currently quiet fellow-eyes (n = 28) (defined by no symptoms of AU for at least 3 months prior to assessment) and in a subgroup of fellow-eyes with no previous uveitis (n = 16) in patients was compared with macular thickness in correspondingly gender- and age matched controls (n = 85, n = 77) by two-sample Student’s t-test.

Inter-ocular differences in macular thickness between AU and their fellow-eyes were compared by HLA-B27 (positive n = 17 versus negative n = 8). Inter-ocular differences were also compared between the initial AU episode (n = 15) and subsequent episodes (n = 9). Both analyses were carried out in scans taken at ≤ 6 weeks from flare-up. A non-parametric test for two independent samples (Mann–Whitney U-test) was used.

SPSS v.17.0 was used for statistical analysis. All tests were two-sided and a p-value < 0.05 was set to be statistically significant.

## Results

Patient demographics and their clinical history and investigations are described in Table 1.

**Table 1:** Patients with anterior uveitis (AU; n = 30): Clinical history and investigations

		**Female**	**Male**	**Total**
**Age**	Mean age (range):	40.7 (19–56)	40.3 (21–69)	
**Duration**	Limited ≤ 3 months	9	14	23
	Persistent > 3 months	6	1	7
**Course**	Acute (sudden onset and limited duration)	9	10	19
	Recurrent (repeated episodes separated by periods of inactivity without treatment ≥3 months in duration)	0	5	5
	Chronic (persistent uveitis with relapse in <3 months after discontinuing treatment)	6	0	6
**Treatment duration**	<1 week	2	0	2
	1-2 weeks	7	12	19
	3-6 weeks	2	2	4
	7-12 weeks	0	1	1
	Chronic (>12 weeks)	3	0	3
	Long term remission	1	0	1
**HLA-B27**	Negative	8	1	9
	Positive	7	14	21
**Relative macular thickening**	Normal (235-259 μm females, 244-272 μm males)	5	3	8
	Thickening (≥259 μm females, ≥272 μm males)	9	12	21
	Atrophy (≤238 μm in females, ≤243 μm males)	1	0	1
**Best corrected visual acuity**	≥0.8	11	13	24
	0.6-0.7	4	0	4
	0.4-0.5	0	1	1
	0.2-0.3	0	1	1
**AU episode**	First episode	7	8	15
	Several episodes	5	7	12
	Chronic	3	0	3
**Anterior chamber cells**	Grade 0, <1 cell	4	3	7
	Grade 0.5+, 1–5 cells	1	2	3
	Grade 1+, 6–15 cells	7	5	12
	Grade 2+, 16–25 cells	3	4	7
	Grade 3+, 26–50 cells	0	1	1
**Spillover**	No retrolental cells	13	9	22
	1-5 retrolental cells	2	6	8
**Systemic symptoms**	Diagnosed spondyloarthropathy	2	9	11
	Joint/ skin/ intestinal/urogenital complaints	4	3	7
	Tiredness, morning stiffness/ pain	6	2	8
	None	3	1	4
**Systemic treatment**	None	11	8	19 (11)*
	Oral prednisolone	2	6	8 (8)*
	Immunomodulatory treatment	2	1	3 (2)*
**Uveitis in contralateral eye**	Never uveitis in contralateral eye	6	10	16 (12)*
	At least one earlier episode	7	5	12 (9)*
	Bilateral uveitis now	2	0	2 (0)*
**Contralateral AU last three month**	Never uveitis	6	10	16
	≤ 3 months prior to examination	1	4	5
	≥ 3 months prior to examination	8	1	9

* The number of HLA-B27+ patients are given in parentheses.

**Figure 2:**
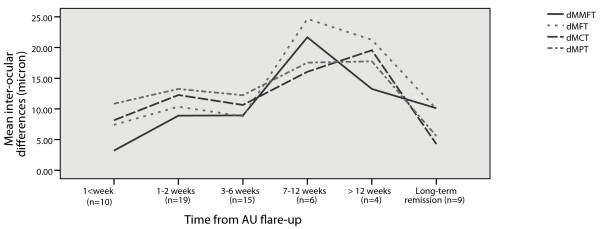
**Mean inter-ocular differences in macular thickness (micron) related to time elapsed from anterior uveitis (AU) flare-up.** dMMFT: mean minimal foveolar thickness, dMFT: mean foveal thickness, dMCT: mean central thickness, dMPT: mean peripheral thickness.

The change in inter-ocular differences in mean macular thickness between affected and fellow-eyes as a function of time elapsed from an AU flare-up in 63 bilateral OCT scans of 29 patients is demonstrated in Figure 2 (one non-repeated scan in 9 patients, 2 repeated scans in 11 patients, 3 repeated scans in 5 patients, 4 repeated scans in 3 patients and 5 repeated scans in one patient). Patients measured late had larger inter-ocular differences in macular thickness: mean differences for MMFT (18.8 μm p = 0.057), MFT (19.4 μm p = 0.068), MCT (13.4 μm p = 0.040), and MPT (9.7 μm p = 0.081).

Macular thickening was observed in 70% of all 30 AU patients; in 79% of patients with acute AU and in 55% of patients with recurrent or chronic AU (Table 1). Maculae in patients were significantly thicker in all regions of AU eyes (p < 0.0005; Table 2).

**Table 2:** Macular thickness(SD) (micron) in AU-affected eyes of patients and controls

**Macular regions/OCT SS**	**Patients (n = 30)**	**Controls (n = 92)**	**Mean differences(SD)**	**p-value**^**1**^
F0 (MMFT)	205(34)	177(20)	28(7)	<0.0005
Inner ring F1(MFT)	237(27)	212(16)	24(5)	<0.0005
Middle ring (MCT)	295(21)	273(15)	22(4)	<0.0005
Outer ring (MPT)	296(20)	274(15)	21(3)	<0.0005
Total region (MTT)	276(21)	253(13)	23(4)	<0.0005
OCT SS	6.2(2)	6.1(2)	0.07	0.85

*AU*: anterior uveitis, *OCT*: Optical coherence tomography, *SS*: signal strength. Five macular areas: *MMFT*: mean minimal foveal thickness, *MFT*: mean foveal thickness, *MCT*: mean central thickness, *MPT*: mean peripheral thickness, *MTT*: mean total thickness. The outer diameter of the inner, middle and outer rings is 1, 2.22 and 3.45 millimeters respectively. For further definition of macular regions see Figure 1. ^1^Two-sample Student's t-test (affected eyes of patients vs. gender-and age matched controls).

Maculae were also thicker in most regions of quiet fellow- eyes compared with gender-and age matched controls (Table 3). Thicker foveola and a trend towards thicker fovea were also observed in a subgroup of patients with no previous uveitis in fellow- eyes (Table 3).

**Table 3:** Macular thickness(SD) (micron) in quiet fellow- eyes of AU patients and controls

**Macular regions/ OCT SS**	**Patients**	**Controls**	**p-value**^**1**^
Currently quiet eyes (contralateral to the AU eye)	(n = 28)^2^	(n = 85)	
F0 (MMFT)	195(27)	179(20)	0.006
Inner ring F1 (MFT)	224(21)	214(15)	0.028
Middle ring MCT	281(17)	274(14)	0.022
Outer ring MPT	281(17)	274(15)	0.057
Total region MTT	262(16)	254(13)	0.024
OCT SS	6.2(2)	6.2(2)	0.76
Quiet eyes with no previously recognized AU	(n = 16)	(n = 77)	
F0 (MMFT)	193(22)	179(20)	0.016
Inner ring F1 (MFT)	223(19)	215(15)	0.092
Middle ring MCT	279(15)	275(14)	0.23
Outer ring MPT	279(16)	275(14)	0.38
Total region MTT	260(15)	255(12)	0.14
OCT SS	5.9(2)	6.1(2)	0.58

*AU*: anterior uveitis, *OCT*: Optical coherence tomography, *SS*: signal strength. Five macular areas: *MMFT*: mean minimal foveolar thickness, *MFT*: mean foveal thickness, *MCT*: mean central thickness, *MPT*: mean peripheral thickness, *MTT*: mean total thickness. The outer diameter of the inner, middle and outer rings is 1, 2.22 and 3.45 millimeters respectively. For further definition of macular regions see Figure 1. ^1^Two- sample Student's t-test (patients vs. gender-and age matched controls). ^2^28 patients with bilateral scans and unilateral uveitis were included in this analysis.

Maculae in AU eyes were thicker than in their fellow- eyes, also in a subgroup of patients with excellent visual acuity (≥0.8) (Table 4).

**Table 4:** Mean differences in macular thickness(SD) (micron) between patients’ affected and fellow- eyes

**Macular regions/ OCT SS**	**Mean differences(SD)**	**T(df) and p-value**^**1**^
**All patients (n = 28)**^2^		
F0 (MMFT)	11.6(21)	2.91(27) 0.007
Inner ring F1 (MFT)	14.1(16)	4.71(27) <0.0005
Middle ring MCT	14.8(12)	6.42(27) <0.0005
Outer ring MPT	15.0(12)	6.83(27) <0.0005
Total region MTT	14.7(12)	6.31(27) <0.0005
OCT SS	−0.04(2)	−0.09(27) 0.93
**Patients with VA ≥ 0.8 (n = 23)**		
F0 (MMFT)	11.6(22)	2.54(22) 0.019
Inner ring F1 (MFT)	14.6(16)	4.42(22) <0.0005
Middle ring MCT	15.5(12)	6.27(22) <0.0005
Outer ring MPT	15.6(11)	6.86(22) <0.0005
Total region MTT	15.3(12)	6.20(22) <0.0005
OCT SS	−0.22(2)	−0.49(22) 0.63

*OCT*: Optical coherence tomography, *SS*: signal strength, *MMFT*: mean minimal foveolar thickness, *MFT*: mean foveal thickness, *MCT*: mean central thickness, *MPT*: mean peripheral thickness, *MTT*: mean total thickness. The outer diameter of the inner, middle and outer rings is 1, 2.22 and 3.45 millimeters respectively. For further definition of macular regions see Figure 1. VA: visual acuity. ^1^One-sample Student's t-test (test value = 0 for differences between patients’ eyes). ^2^28 patients with bilateral scans were included in this analysis.

**Figure 3:**
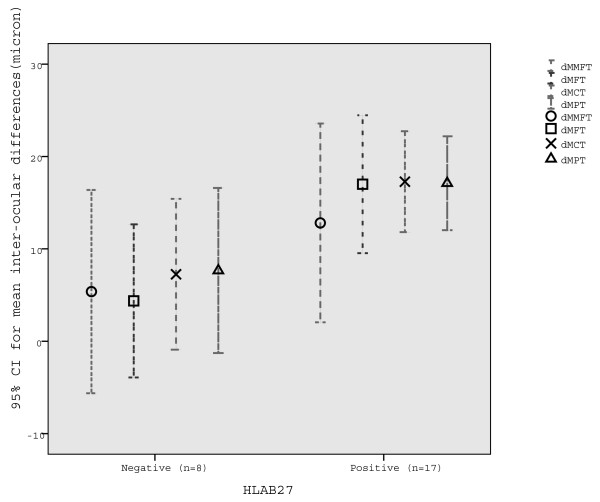
**Mean inter-ocular differences (and 95% confidence intervals) (micron) by HLA-B27 in anterior uveitis.** dMMFT: mean minimal foveolar thickness, dMFT: mean foveal thickness, dMCT: mean central thickness, dMPT: mean peripheral thickness.

The majority of our patients were HLA-B27-positive (70%), they had acute AU (63%) which was of limited duration (77%). Most patients had mild anterior chamber inflammation (grade ≤1+) (73%) and excellent VA (≥0.8) (80%) when scans were taken (Table 1). Inter-ocular differences in macular thickness were larger in HLAB-27-positive than in HLAB-27-negative patients (on scans taken at ≤6 weeks from AU flare-up); MMFT: p = 0.43 MFT: p = 0.083 MCT: p = 0.032 MPT: p = 0.025 MTT: p = 0.011 (exact two-sided p-values). Mean inter-ocular differences are shown in Figure 3.

Inter-ocular differences were compared between patients in which the current AU was their initial episode (n = 15) with those in which the episode was subsequent (n = 9) (on scans taken at ≤6 weeks from AU flare-up). Differences were small and did not achieve the set statistical significance; MMFT: p = 0.27, MFT: p = 0.44, MCT: p = 0.83, MPT: p = 0.69, MMT: p = 0.77) (exact two-sided p-values).

## Discussion

In the present study we report a new observation: macular thickening is present in quiet fellow-eyes of AU patients, also in the foveola of fellow-eyes with no previous uveitis. This finding suggests that an AU episode is accompanied by a systemic immune-mediated response which affects both patients’ eyes. Another important finding in the present study was that MT is rather the rule than the exception even in phakic AU eyes with good VA, suggesting that some extent of relative MT accompanies most, even uncomplicated AU cases. In addition, MT seems to be larger in HLA-B27-related than in HLA-B27-negative AU, suggesting a stronger subclinical involvement of the posterior segment in HLA-B27-positive patients, which is probably not related to recurrence. These observations are in line with and extend the current understanding of the mechanisms behind MT in AU. It is assumed that they are similar to those involved in uveitic macular edema. It is thought that trans-cellular mechanisms along with a functioning blood-retina barrier regulate the fluid equilibrium in the retina [31]. Biologically active substances related to inflammation together with other predisposing factors may compromise the functional integrity of this barrier with a consequential MT in uveitis [32,33].

MT has been previously described in eyes with chronic or severe AU [11,34] and in AU with a broader range of inflammation severity [13]. The latter study reported an overall rate of MT in 42% of AU patients, although eyes with pseudophakia and cystoid macular edema were included. Since cataract surgery and cystoid space formation may contribute to MT [10,35,36], we expected to find lower rates of MT in our sample, from which eyes with psedophakia and detectable cystoid space formation were excluded. In addition our definition of MT was strict and gender- adjusted. Nonetheless MT was observed in 70% of AU eyes in our sample. Our data suggest that MT is rather the rule than the exception even in phakic eyes with excellent VA.

Besides the possible genuine differences in the rate and degree of MT between Castellano et al.’s [13] and our samples, the measured differences may also be attributed to dissimilar time intervals between the AU flare-up and OCT examination. Castellano et al. [13] included only OCT scans taken on first visit, although 56% of their patients had chronic AU. Our data may indicate that MT lags several weeks behind the clinical AU flare-up, which is coherent with Traill et al.’s assumption [11]. Hegde et al. [37] observed no MT in patients with acute AU of ≤ 1 week duration, and it seems that MT peaks at about 6–12 weeks after the initial flare-up, often after a clinical resolution of the AU.

For these reasons we assume that macular thickness increases with time elapsed from the initial flare-up, although the statistical evidence for this assumption is rather weak. Individual clinically tailored follow-up intervals were not optimal for the statistical analyses, which eventually resulted in a small study sample for the “late” follow-up group. However, Peizeng et al. [38] detected inflammatory changes in the ciliary body on ultrasound at 6 weeks follow-up in acute AU in spite of absence of inflammatory anterior chamber cells. It is accordingly possible that a continuous release of inflammatory mediators from the ciliary body can explain the time lag.

We observed that differences in macular thickness between affected eyes and fellow-eyes of patients were smaller than differences between patient’s affected eyes and controls. This observation can be explained if the increase in macular thickness after a previous AU episode lasts for at least 3 months (n = 12). However, the duration of residual MT after an AU episode has not been established. Traill et al. found MT in 45% of eyes at 6 months [11] while Moreno-Arrones et al. found no MT at 6.3 months [16]. Bilateral AU (n = 1 in our sample) will decrease the inter-eye MT difference.

Subclinical AU in some fellow-eyes should also be considered. Indeed central maculae in fellow-eyes with no previously diagnosed uveitis (n = 16) were thicker than in controls. We did not find any literature dealing with macular thickness in unaffected fellow-eyes during an uveitis episode. We do not know the reason for this observation. However activation of the peripheral immunoregulatory mechanisms and increased levels of ocular autoantigens have been found in AU patients [39,40]. Furthermore, up-regulated levels of IL-22 which may compromise the blood-retina barrier and allow biologically active substances and water to enter the retina were found in peripheral blood in uveitis [41,42]. We hypothesize that similar mechanisms may also influence fellow-eyes of AU patients, explaining the simultaneous increase in MT in both eyes of patients even when AU is unilateral.

Posterior segment involvement in HLA-B27-related uveitis is not uncommon, and it is probably under-recognized [43-45]. Power et al. [23] reported that cystoid macula edema was five times more frequent in HLA-B27-positive than HLA-B27-negative AU, the occurrence of which may predict a less favorable visual outcome [46]. However, Castellano et al. [13] found no differences in MT by HLA-B27 on patients’ first visit. We observed a larger MT in HLA-B27-related than in HLA-B27-negative AU. It is possible that early MT is independent of the antigen, while the magnitude of further increase is related to both the HLA-B27 and/or its polymorphism, along with the time perspective discussed above. It is accordingly possible that HLA-B27-related AU is accompanied by a larger or a more prolonged MT than HLA-B27-negative AU.

We wonder whether the above described dispute about the duration of residual MT after an AU episode [11,13,16] is partly related to different HLA-B27- antigen distribution in studied samples; Traill et al.’s sample was dominated by HLA-B27-positive patients (as the case is with our sample, while only 42% of Castellano et al.’s sample are HLA-B27-positive. Moreno-Arronez et al. do not mention the distribution of HLA-B27 antigen in their sample.

The absence of cystoid spaces and detectable epiretinal membrane is consistent with good VA [8,10] which probably carries a beneficial long-term visual prognosis for patients in our sample. Markomichelakis et al. [8] demonstrated complete resolution of uveitic edema in 47% of eyes by 12 months follow-up with medical treatment. They suggested that diffuse MT could carry a negative prognostic significance because it was not treated due to good VA. However, diffuse MT may adversely affect visual function on persistence or during inflammatory relapse [47]. We did not observe differences in MT on recurrence. However, other aspects of visual function such as central visual field impairment may occur even on complete resolution of edema despite good VA [48,49]. The prognostic relevance of MT on long-term visual function in AU is not yet known.

The present study has some limitations. Although hospital records were examined in all cases, the previous history depended on patients’ recollection ability in some cases.

Follow-up intervals were individually tailored depending on the AU severity, treatment intensity and response, as well as practicability for ophthalmologist and patient. Intervals were therefore not standardized for the purpose of statistical analyses. In order to demonstrate an increase in thickness as a function of time elapsed from AU flare-up, mean inter-ocular differences were averaged from different cases at different intervals in lack of sufficient repeated longitudinal data. Longer follow-up intervals could preferentially select the more severe or longer-lasting cases, biasing the time frame towards larger measured thickness (if MT is related to severity and/or chronicity, which we do not know) in our small study sample. However, according to medical records this is not the case. Nevertheless, these data should be interpreted with care until confirmed in larger samples.

Three patients had been receiving an immunomodulatory agent for rheumatic disease other than uveitis before current AU flare-up. Eight patients received a short course of oral prednisolone during current AU episode. Prednisolone alone or in combination with other immunomodulatory agents is the mainstay treatment in uveitis and uveitic edema [32,33,50,51]. Immunomodulatory agents may even reduce frequency of recurrence [52,53]. Thus systemic treatment these 11 patients received may have protected their retinae from further thickening. Macular thickness may have otherwise reached even greater values than observed in this study.

## Conclusions

Subclinical MT is present in both affected eyes and quiet fellow-eyes of AU-patients. MT is detected by OCT during most cases of AU, even in phakic eyes with good VA and in quiet fellow-eyes with no previous uveitis. MT probably reflects systemic immune-mediated response to the inflammatory disorder in AU, and it is possible that HLA-B27-related factors are involved in the pathogenesis of AU. These observations are in line with and extend the current understanding of the mechanisms behind MT in AU.

## Competing interests

The authors declare that they have no competing interests.

## Authors’ contributions

TBE suggested the initial concept and together with AW was responsible for planning the study design. AW carried out the data collection including OCT measurements. TS helped with the statistical analysis and interpretation. All authors have participated in the writing and approval of the final manuscript.

## Pre-publication history

The pre-publication history for this paper can be accessed here:

http://www.biomedcentral.com/1471-2415/12/30/prepub

## References

[B1] Hooper C McCluskey P Intraocular inflammation: Its causes and investigations Curr Allergy Asthma Rep 2008 8 4 331 338 10.1007/s11882-008-0053-3 18606087

[B2] Jabs DA Nussenblatt RB Rosenbaum JT Standardization of uveitis nomenclature for reporting clinical data. Results of the first international workshop Am J Ophthalmol 2005 140 3 509 516 16196117 10.1016/j.ajo.2005.03.057 PMC8935739

[B3] Rothova A Suttorp-van Schulten MS Frits Treffers W Kijlstra A Causes and frequency of blindness in patients with intraocular inflammatory disease Br J Ophthalmol 1996 80 4 332 336 10.1136/bjo.80.4.332 8703885 PMC505460

[B4] Durrani OM Tehrani NN Marr JE Moradi P Stavrou P Murray PI Degree, duration, and causes of visual loss in uveitis Br J Ophthalmol 2004 88 9 1159 1162 10.1136/bjo.2003.037226 15317708 PMC1772296

[B5] Lardenoye CW van Kooij B Rothova A Impact of macular edema on visual acuity in uveitis Ophthalmology 2006 113 8 1446 1449 10.1016/j.ophtha.2006.03.027 16877081

[B6] Maini R O'Sullivan J Reddy A Watson S Edelsten C The risk of complications of uveitis in a district hospital cohort Br J Ophthalmol 2004 88 4 512 517 10.1136/bjo.2002.013334 15031168 PMC1772087

[B7] Chang JHM Wakefield D Uveitis: A global perspective Ocul Immunol Inflamm 2002 10 4 263 279 10.1076/ocii.10.4.263.15592 12854035

[B8] Markomichelakis NN Halkiadakis I Pantelia E Peponis V Patelis A Theodossiadis P Theodossiadis G Patterns of macular edema in patients with uveitis: Qualitative and quantitative assessment using optical coherence tomography Ophthalmology 2004 111 5 946 953 10.1016/j.ophtha.2003.08.037 15121373

[B9] Estafanous MF Lowder CY Kaiser PK Patterns of macular edema in uveitis patients Ophthalmology 2005 112 2 360 author reply 360–361 15691581 10.1016/j.ophtha.2004.11.011

[B10] Iannetti L Accorinti M Liverani M Caggiano C Abdulaziz R Pivetti-Pezzi P Optical coherence tomography for classification and clinical evaluation of macular edema in patients with uveitis Ocul Immunol Inflamm 2008 16 4 155 160 10.1080/09273940802187466 18716950

[B11] Traill A Stawell R Hall A Zamir E Macular thickening in acute anterior uveitis Ophthalmology 2007 114 2 402 10.1016/j.ophtha.2006.07.028 17270703

[B12] Brown JC Solomon SD Bressler SB Schachat AP DiBernardo C Bressler NM Detection of diabetic foveal edema: Contact lens biomicroscopy compared with optical coherence tomography Arch Ophthalmol 2004 122 3 330 335 10.1001/archopht.122.3.330 15006844

[B13] Castellano CG Stinnett SS Mettu PS McCallum RM Jaffe GJ Retinal thickening in iridocyclitis Am J Ophthalmol 2009 148 3 341 349 10.1016/j.ajo.2009.03.034 19477710

[B14] Akduman L Can we be more objective in determining the response to treatment in uveitis patients aside from recording anterior chamber reaction? Ocul Immunol Inflamm 2009 17 4 265 266 10.1080/09273940802702587 19657980

[B15] Ducos de Lahitte G Terrada C Tran TH Cassoux N LeHoang P Kodjikian L Bodaghi B Maculopathy in uveitis of juvenile idiopathic arthritis: An optical coherence tomography study Br J Ophthalmol 2008 92 1 64 69 10.1136/bjo.2007.120675 17585000

[B16] Moreno-Arrones JP Gorrono-Echebarria MB Teus-Guezala MA Macular thickening in acute anterior uveitis with a 6-month remission period Canadian Journal of Ophthalmology-Journal Canadien D Ophtalmologie 2010 45 1 91 92 20130729 10.3129/i09-195

[B17] Braakenburg AM de Valk HW de Boer J Rothova A Human leukocyte antigen-b27-associated uveitis: Long-term follow-up and gender differences Am J Ophthalmol 2008 145 3 472 479 10.1016/j.ajo.2007.11.009 18282492

[B18] Zeboulon N Dougados M Gossec L Prevalence and characteristics of uveitis in the spondyloarthropathies: A systematic literature review Ann Rheum Dis 2008 67 7 955 959 17962239 10.1136/ard.2007.075754

[B19] Khan MA Hla-b27 and its subtypes in world populations Curr Opin Rheumatol 1995 7 4 263 269 10.1097/00002281-199507000-00001 7547102

[B20] Khan MA Mathieu A Sorrentino R Akkoc N The pathogenetic role of hla-b27 and its subtypes Autoimmun Rev 2007 6 3 183 189 10.1016/j.autrev.2006.11.003 17289555

[B21] Chang JH McCluskey PJ Wakefield D Acute anterior uveitis and hla-b27 Surv Ophthalmol 2005 50 4 364 388 10.1016/j.survophthal.2005.04.003 15967191

[B22] Sheehan NJ Hla-b27: What's new? Rheumatology (Oxford) 2010 49 4 621 631 10.1093/rheumatology/kep450 20083539

[B23] Power WJ Rodriguez A Pedroza-Seres M Foster CS Outcomes in anterior uveitis associated with the hla-b27 haplotype Ophthalmology 1998 105 9 1646 1651 10.1016/S0161-6420(98)99033-9 9754172

[B24] Rothova A van Veenedaal WG Linssen A Glasius E Kijlstra A de Jong PT Clinical features of acute anterior uveitis Am J Ophthalmol 1987 103 2 137 145 3492916 10.1016/s0002-9394(14)74218-7

[B25] Gran JT Mellby AS Husby G The prevalence of hla-b27 in northern norway Scand J Rheumatol 1984 13 2 173 176 10.3109/03009748409100382 6610933

[B26] Bakland G Nossent HC Gran JT Incidence and prevalence of ankylosing spondylitis in northern norway Arthritis Rheum 2005 53 6 850 855 10.1002/art.21577 16342091

[B27] Blochmichel E Nussenblatt RB International uveitis study group recommendations for the evaluatioon of intraocular inflammatory disease Am J Ophthalmol 1987 103 2 234 235 3812627 10.1016/s0002-9394(14)74235-7

[B28] Deschenes J Murray PI Rao NA Nussenblatt RB International uveitis study group (iusg) clinical classification of uveitis Ocul Immunol Inflamm 2008 16 1–2 1 2 18379933 10.1080/09273940801899822

[B29] Lyon F Gale RP Lightman S Recent developments in the treatment of uveitis: An update Expert Opin Investig Drugs 2009 18 5 609 616 10.1517/14728220902852570 19388878

[B30] Wexler A Sand T Elsas TB Macular thickness measurements in healthy norwegian volunteers: An optical coherence tomography study BMC Ophthalmol 2010 10 1 13 10.1186/1471-2415-10-13 20465801 PMC2885325

[B31] Marmor MF Mechanisms of fluid accumulation in retinal edema Doc Ophthalmol 1999 97 3–4 239 249 10896337 10.1023/a:1002192829817

[B32] Okhravi N Lightman S Cystoid macular edema in uveitis Ocul Immunol Inflamm 2003 11 1 29 38 10.1076/ocii.11.1.29.15582 12854025

[B33] Rothova A Inflammatory cystoid macular edema Curr Opin Ophthalmol 2007 18 6 487 492 10.1097/ICU.0b013e3282f03d2e 18163001

[B34] Brar M Yuson R Kozak I Mojana F Cheng L Bartsch DU Oster SF Freeman WR Correlation between morphologic features on spectral-domain optical coherence tomography and angiographic leakage patterns in macular edema Retina 2010 30 3 383 389 10.1097/IAE.0b013e3181cd4803 20216291 PMC2870721

[B35] Biro Z Balla Z Kovacs B Change of foveal and perifoveal thickness measured by oct after phacoemulsification and iol implantation Eye 2008 22 1 8 12 10.1038/sj.eye.6702460 16751754

[B36] Kecik D Makowiec-Tabernacka M Golebiewska J Moneta-Wielgos J Kasprzak J Macular thickness and volume after uncomplicated phacoemulsification surgery evaluated by optical coherence tomography. A one-year follow-up Neuro Endocrinol Lett 2009 30 5 610 614 20035257

[B37] Hegde V Pagliarini S Macular analysis with optical coherence tomography (oct-3) and its role as a screening tool in acute anterior uveitis Invest Ophthalmol Vis Sci 2004 45 U989 U989

[B38] Peizeng Y Qianli M Xiangkun H Hongyan Z Li W Kijlstra A Longitudinal study of anterior segment inflammation by ultrasound biomicroscopy in patients with acute anterior uveitis Acta Ophthalmol 2009 87 2 211 215 10.1111/j.1755-3768.2008.01194.x 18811638

[B39] Deschenes J Char DH Kaleta S Activated t lymphocytes in uveitis Br J Ophthalmol 1988 72 2 83 87 10.1136/bjo.72.2.83 2964862 PMC1041377

[B40] Heiligenhaus A Rebmann V Neubert A Plewa S Ferencik S Vogeler U Steuhl KP Grosse-Wilde H Soluble hla class i and hla-dr plasma levels in patients with anterior uveitis Tissue Antigens 2004 63 4 369 375 10.1111/j.0001-2815.2004.00201.x 15009809

[B41] Li Z Liu B Maminishkis A Mahesh SP Yeh S Lew J Lim WK Sen HN Clarke G Buggage R Gene expression profiling in autoimmune noninfectious uveitis disease J Immunol 2008 181 7 5147 5157 18802119 10.4049/jimmunol.181.7.5147 PMC2631441

[B42] Kebir H Kreymborg K Ifergan I Dodelet-Devillers A Cayrol R Bernard M Giuliani F Arbour N Becher B Prat A Human th17 lymphocytes promote blood–brain barrier disruption and central nervous system inflammation Nat Med 2007 13 10 1173 1175 10.1038/nm1651 17828272 PMC5114125

[B43] Uy HS Christen WG Foster CS Hla-b27-associated uveitis and cystoid macular edema Ocul Immunol Inflamm 2001 9 3 177 183 10.1076/ocii.9.3.177.3963 11815886

[B44] Dodds EM Lowder CY Meisler BM Posterior segment inflammation in hla-b27+acute anterior uveitis: Clinical characteristics Ocul Immunol Inflamm 1999 7 2 85 92 10.1076/ocii.7.2.85.4015 10420203

[B45] Rodriguez A Akova YA Pedrozaseres M Foster CS Posterior segment ocular manifestations in patients with hla-b27-associated uveitis Ophthalmology 1994 101 7 1267 1274 8035991 10.1016/s0161-6420(94)31179-1

[B46] Rothova A Medical treatment of cystoid macular edema Ocul Immunol Inflamm 2002 10 4 239 246 10.1076/ocii.10.4.239.15589 12854032

[B47] Markomichelakis NN Halkiadakis I Pantelia E Georgalas E Anthi K Theodossiadis P Moschos M Theodossiadis G Kouvatseas G Course of macular edema in uveitis under medical treatment Ocul Immunol Inflamm 2007 15 2 71 79 10.1080/09273940701244509 17558831

[B48] Kiss CG Barisani-Asenbauer T Simader C Maca S Schmidt-Erfurth U Central visual field impairment during and following cystoid macular oedema Br J Ophthalmol 2008 92 1 84 88 10.1136/bjo.2007.124016 17591669

[B49] Paroli MP Spinucci G Fabiani C Pivetti-Pezzi P Retinal complications of juvenile idiopathic arthritis-related uveitis: A microperimetry and optical coherence tomography study Ocul Immunol Inflamm 2010 18 1 54 59 10.3109/09273940903311999 20128652

[B50] Lim L Suhler EB Smith JR Biologic therapies for inflammatory eye disease Clin Experiment Ophthalmol 2006 34 4 365 374 10.1111/j.1442-9071.2006.01225.x 16764659

[B51] Sharma SM Nestel AR Lee RWJ Dick AD Clinical review: Anti-tnf alpha therapies in uveitis: Perspective on 5 years of clinical experience Ocul Immunol Inflamm 2009 17 6 403 414 10.3109/09273940903072443 20001261

[B52] Braun J Baraliakos X Listing J Sieper J Decreased incidence of anterior uveitis in patients with ankylosing spondylitis treated with the anti-tumor necrosis factor agents infliximab and etanercept Arthritis Rheum 2005 52 8 2447 2451 10.1002/art.21197 16052578

[B53] Sieper J Koenig A Baumgartner S Wishneski C Foehl J Vlahos B Freundlich B Analysis of uveitis rates across all etanercept ankylosing spondylitis clinical trials Ann Rheum Dis 2010 69 1 226 229 10.1136/ard.2008.103192 19465402

